# Supplementary material for: Neutrophil microvesicles drive atherosclerosis by delivering miR-155 to atheroprone endothelium
Source: Nat Commun. 2020 Jan 10;11:214. doi: 10.1038/s41467-019-14043-y (PMC6954269; doi:10.1038/s41467-019-14043-y)
Supplement: Supplementary file 2 — Supplementary Information [file 41467_2019_14043_MOESM2_ESM.pdf]

Neutrophil microvesicles drive atherosclerosis by delivering *miR-155* to atheroprone endothelium.

Gomez *et al*

SUPPLEMENTARY INFORMATION

Supplementary Figures

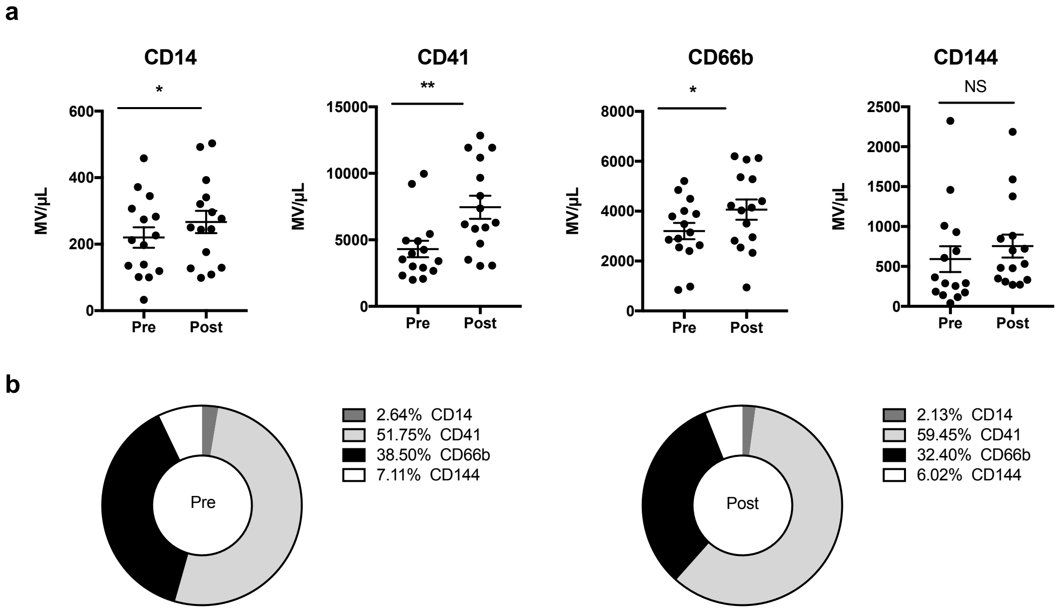

Supplementary Figure 1. Circulating MV numbers in human plasma pre and post high fat diet. MVs present in human plasma samples taken pre and post one week high fat diet were incubated with fluorescently-labelled antibodies to cell-specific markers: CD14 (monocytes); CD41 (platelets); CD66b (neutrophils); CD144 (endothelial cells), n = 15 subjects. In order to quantify the MVs, Sphero™AccuCount counting beads were added to each sample prior to analysis. The number of MVs (**a**) and percentage of total MVs (**b**) within the positive gate (according to the gating strategies shown in Supplementary Figure 1e) was quantified using flow cytometry and analyzed using FlowJo analysis software. Data are presented as mean ± SEM and statistical significance evaluated using a paired *t*-test assuming a Gaussian distribution. * = $P < 0.05$, ** = $P < 0.01$, NS = not significant. Source data are provided as a Source Data file.

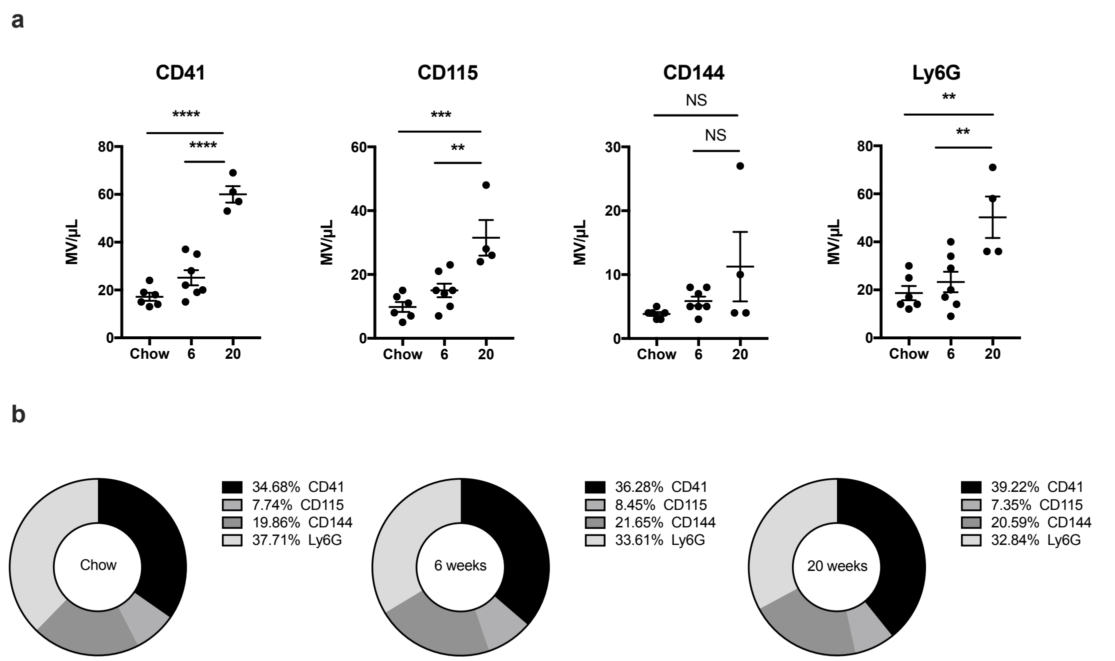

Supplementary Figure 2. MV numbers in mouse aortic arch homogenate. MVs present in aortic arch homogenates prepared from *ApoE*$^{-/-}$ mice on chow for 20 weeks (n = 6 animals) or Western diet for 6 (n = 6 animals) or 20 weeks (n = 4 animals) were incubated with fluorescently-labelled antibodies to cell-specific markers: CD41 (platelets); CD115 (monocytes); CD144 (endothelial cells) and Ly6G (neutrophils). In order to quantify the MVs, Sphero™AccuCount counting beads were added to each sample prior to analysis. The number of MVs (**a**) and percentage of total MVs (**b**) within the positive gate (according to the gating strategies shown in Supplementary Figure 2) was quantified using flow cytometry and analyzed using FlowJo analysis software. Data are presented as mean ± SEM and statistical significance evaluated using a one-way ANOVA followed by Tukey's test for multiple comparisons. ** = $P < 0.01$, *** = $P < 0.001$, **** = $P < 0.0001$, NS = not significant. Source data are provided as a Source Data file.

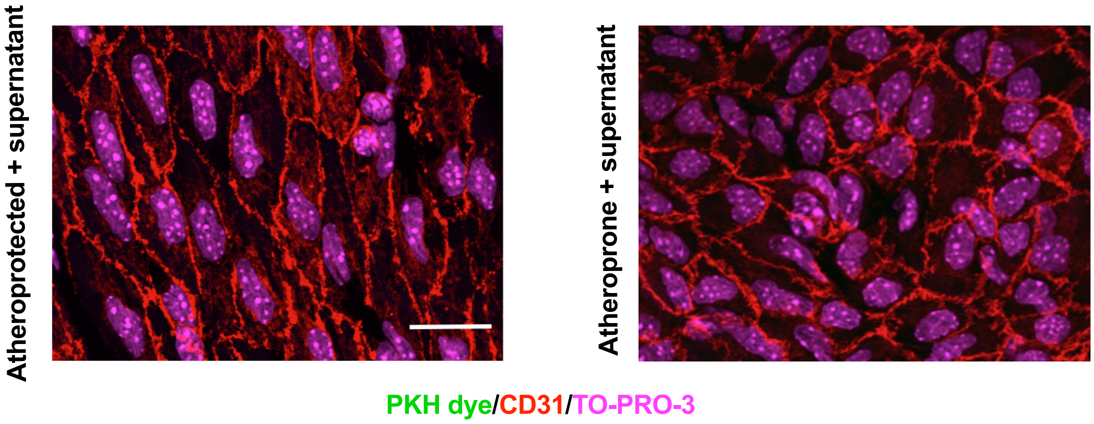

Supplementary Figure 3. Supernatant control for *in vivo* NMV adhesion. Supernatants from preparations of fluorescently-labelled NMVs (green) were injected via the tail vein into *ApoE*$^{-/-}$ mice that had been fed a Western diet for 6 weeks. After 2 h, mice were culled and *en face* immunostaining of the mouse aortic arch was performed. Representative *en face* images of atheroprotected (outer curvature) and atheroprone (inner curvature) regions of the aorta, visualized by confocal fluorescence microscopy are shown. Scale bar = 20 µm. Endothelial cells were identified by staining with anti-CD31 antibody (red) and cell nuclei were identified using TO-PRO Iodide (magenta). Outer and inner curvature of the ascending aorta were identified by anatomical landmarks and confirmed by characterising the phenotype of endothelial cells; those at the outer curvature were aligned, elongated and uniform – a characteristic of cells under high shear, whereas cells in the inner curvature had a disorganized appearance.

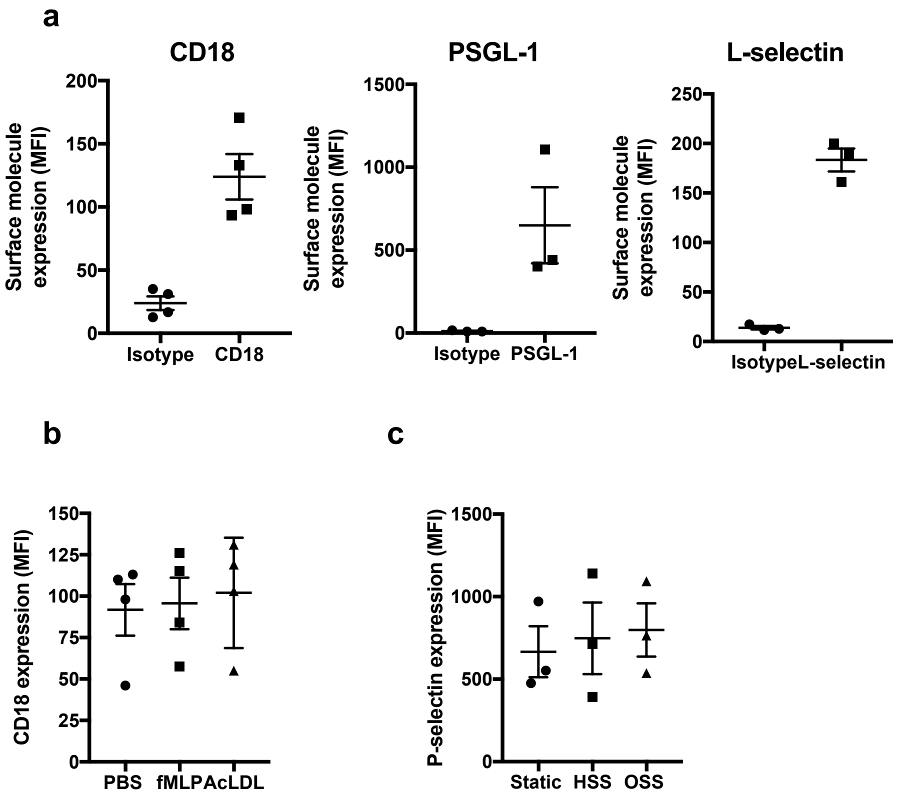

Supplementary Figure 4. Adhesion molecule analysis. (**a**) To analyze CD18, PSGL-1 and L-selectin expression, NMVs were incubated with fluorescently labelled anti-CD18, -PSGL-1 or –L-selectin antibodies or isotype control and mean fluorescence intensity (MFI) analyzed using flow cytometry. Data are represented as mean ± SEM (n = 4 independent experiments). (**b**) CD18 expression on the surface of NMVs released by neutrophils incubated with PBS (with calcium and magnesium), fMLP (10 µmol L^{-1}) or acLDL (20 µg mL^{-1}) for 1h at 37 °C was also analyzed by flow cytometry. Data are represented as mean ± SEM (n = 4 independent experiments). (**c**) HCAEC cultured for 72 h under HSS or OSS were incubated with fluorescently labelled anti-P-selectin antibody and MFI was analyzed using flow cytometry. Data are represented as mean ± SEM (n = 3 independent experiments) and statistical significance evaluated using one-way ANOVA followed by Tukey's post hoc test for multiple comparisons. Source data are provided as a Source Data file.

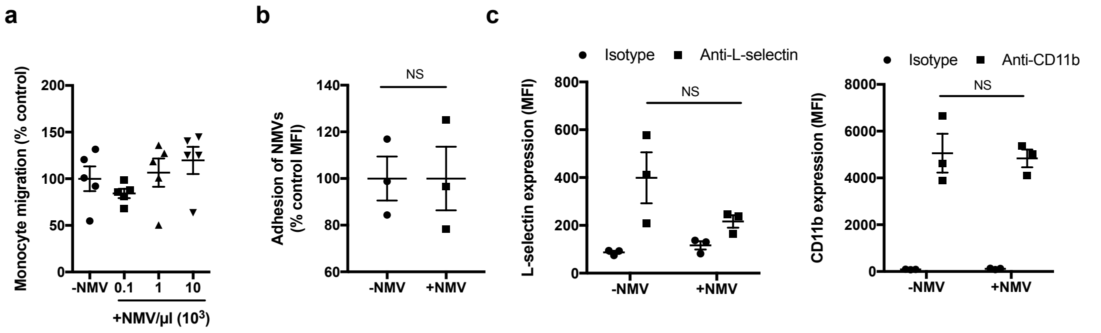

Supplementary Figure 5. NMV interactions with monocytes. (**a**) HCAEC were cultured on transwell inserts and incubated with (+NMV) or without (-NMV) NMVs from unstimulated neutrophils for 30 min followed by addition of monocytes. The number of monocytes that transmigration into the lower chamber in response to CCL2 (5 nmol L^{-1}) was analyzed after 90 min (n = 5 independent experiments). Data are represented as mean ± SEM (n = 5) and analyzed for statistical significance using a one-way ANOVA followed by Dunnett's comparing all samples to -NMV control. (**b**) Fluorescently labelled NMVs were incubated with monocytes for 2 h at 37 °C. Unbound NMVs were removed and the cells were washed. Changes in mean fluorescence intensity (MFI) were measured using flow cytometry to determine NMV adhesion to monocytes Data are represented as mean ± SEM mean (n = 3) and analyzed for statistical significance using a paired *t*-test assuming a Gaussian distribution. (**c**) Monocytes were incubated with (+NMV) or without (-NMV) NMVs for 30 min, washed and stained with fluorescently labelled anti-L-selectin or anti-CD11b or their respective isotype controls. Changes in mean fluorescence intensity (MFI) were measured using flow cytometry to analyze surface adhesion molecule expression. Data are represented as mean ± SEM (n = 3 independent experiments) and statistical significance evaluated using a two-way ANOVA followed by Tukey's post hoc test. NS = not significant. Source data are provided as a Source Data file.

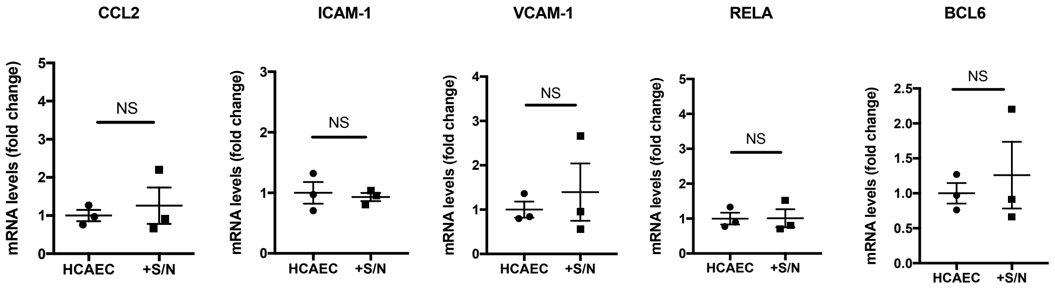

Supplementary Figure 6. Endothelial cell responses to NMV supernatant. HCAEC were incubated with (+S/N) or without (HCAEC) supernatants taken from NMV pellets for 2 h and alterations in inflammatory gene expression investigated using RT-qPCR (n = 3 independent experiments). Samples were normalized to β-actin. Data are represented as mean ± SEM and statistical significance evaluated using a paired *t*-test assuming a Gaussian distribution. Source data are provided as a Source Data file.

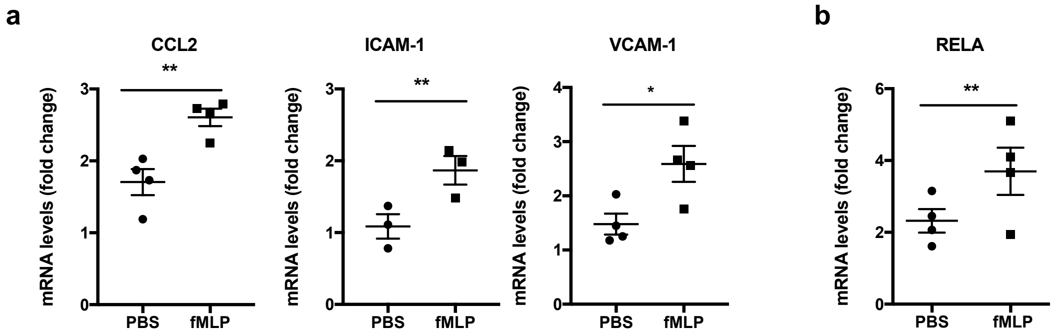

Supplementary Figure 7. Endothelial cell responses to NMVs released by unstimulated neutrophils. (**a** - **b**) HCAEC were incubated with NMVs from unstimulated (PBS) or stimulated (fMLP) neutrophils for 2 h and (**a**) alterations in inflammatory gene expression investigated using RT-qPCR (n = 4 independent experiments). (**b**) *RELA* gene expression was measured using RT-qPCR (n = 4 independent experiments). Samples normalized with β-actin and are expressed as fold change compared to gene expression levels in unstimulated HCAEC. Data are represented as mean ± SEM and statistical significance evaluated using a paired *t*-test assuming a Gaussian distribution. * $P < 0.05$, ** $P < 0.01$. Source data are provided as a Source Data file.

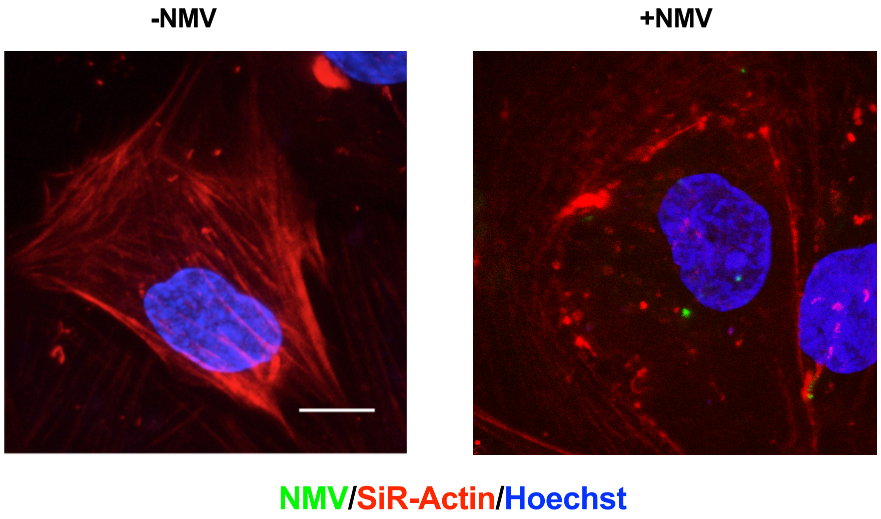

Supplementary Figure 8. Supernatant control for *in vitro* NMV internalisation experiments. Representative Z-stack maximal intensity projection of HCAEC incubated with supernatants from preparations of fluorescently-labelled NMVs (-NMV; green) or fluorescently-labelled NMVs (+NMV; green) and stained with SiR-Actin (F-actin; red) and Hoechst (nuclei; blue). Scale bar = 10 µm.

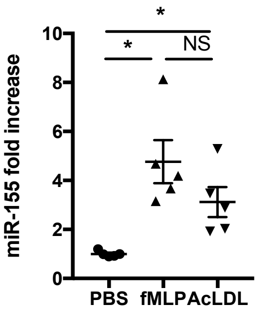

Supplementary Figure 9. Relative *miR-155* content of MVs derived from neutrophils exposed to different stimuli. The *miR-155* content of 2 × 10^6 NMVs prepared from human peripheral blood neutrophils incubated with PBS (with calcium and magnesium), fMLP (10 µmol L^{-1}) or acLDL (20 µg mL^{-1}) for 1h at 37 °C was quantified by RT-qPCR (n = 5 indpendent experiments). Data are represented as mean fold increase ± SEM compared to NMVs from unstimulated (PBS) neutrophils and statistical significance evaluated using a one-way ANOVA followed by Tukey's test for multiple comparisons. * $P < 0.05$, *** $P < 0.001$. Source data are provided as a Source Data file.

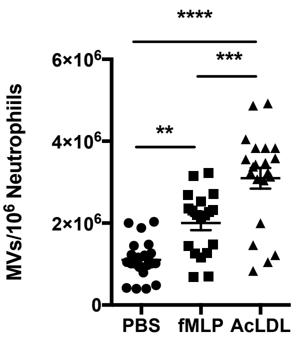

Supplementary Figure 10. Quantification of MV release from neutrophils exposed to different stimuli. Isolated human peripheral blood neutrophils (1×10^6 mL^{-1}) were incubated with PBS (with calcium and magnesium, n= 23), fMLP (10 µmol L^{-1}, n = 19) or acLDL (20 µg mL^{-1}, n = 21) for 1h at 37 °C. Samples were then cleared of cells and large cellular debris by centrifugation and Sphero™AccuCount counting beads were added to each sample prior to analysis. The number of MVs was quantified using flow cytometry and analyzed using FlowJo analysis software. Data are presented as mean ± SEM and statistical significance evaluated using one-way ANOVA followed by Tukey's test for multiple comparisons. ** = $P < 0.01$, *** = $P < 0.001$, **** = $P < 0.0001$. Source data are provided as a Source Data file. n numbers represent biologically independent experiments.

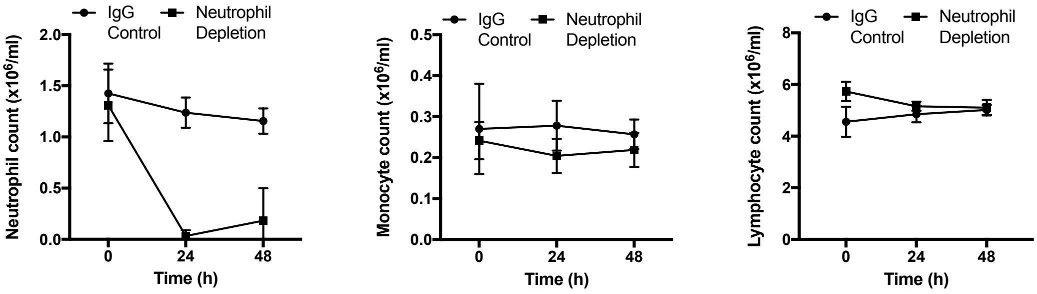

Supplementary Figure 11. Analysis of circulating leukocyte levels after anti-Ly6G antibody administration. *ApoE*$^{-/-}$ mice were injected with anti-Ly6G antibody (100 µg per injection) and total and differential cell counts performed on blood samples taken after termination of the experiment at the times indicated. Data are expressed as mean ± SEM, n = 3 independent animals per time point. Source data are provided as a Source Data file.

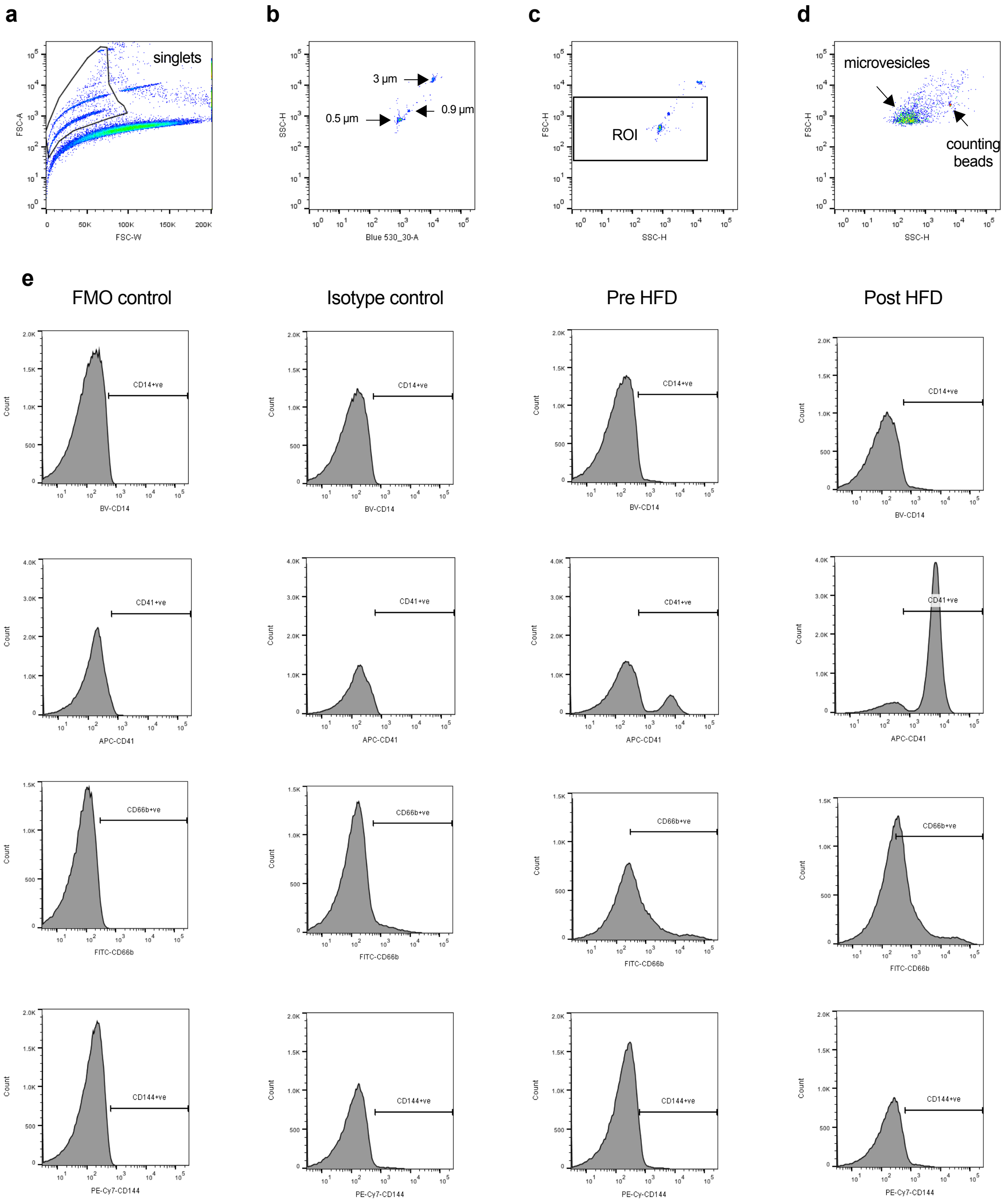

Supplementary Figure 12. Quantification of MVs and gating strategy for human plasma MV analysis. LSRII flow cytometer settings were standardized using Megamix beads. (**a**) The singlet population was defined using forward scatter area (FSC-A) versus forward scatter width (FSC-W). (**b**) Bead subsets were identified based on side scatter height (SSC-H) and fluorescence properties and (**c**) using their forward scatter height (FSC-H) and side scatter height (SSC-H) a region of interest (ROI) marked around the 0.5 and 0.9 µm bead subsets for orientation. (**d**) Once these parameters were set, samples containing Sphero™AccuCount beads of a known concentration for quantification were analyzed. (**e**) Representative flow cytometry histogram plots showing gating strategy for the identification of $CD14^{+}$, $CD41^{+}$, $CD66b^{+}$ and $CD144^{+}$ MVs in human samples (log scale). Fluorescence minus one samples were used to set the positive gate and isotype control samples were analyzed to ensure the signal was due to specific binding of the antibody to the surface marker. Human plasma samples taken pre and post high fat diet were then incubated with antibodies to all markers. In order to quantify the microvesicles, Sphero™AccuCount counting beads were added to each sample prior to analysis on the flow cytometer.

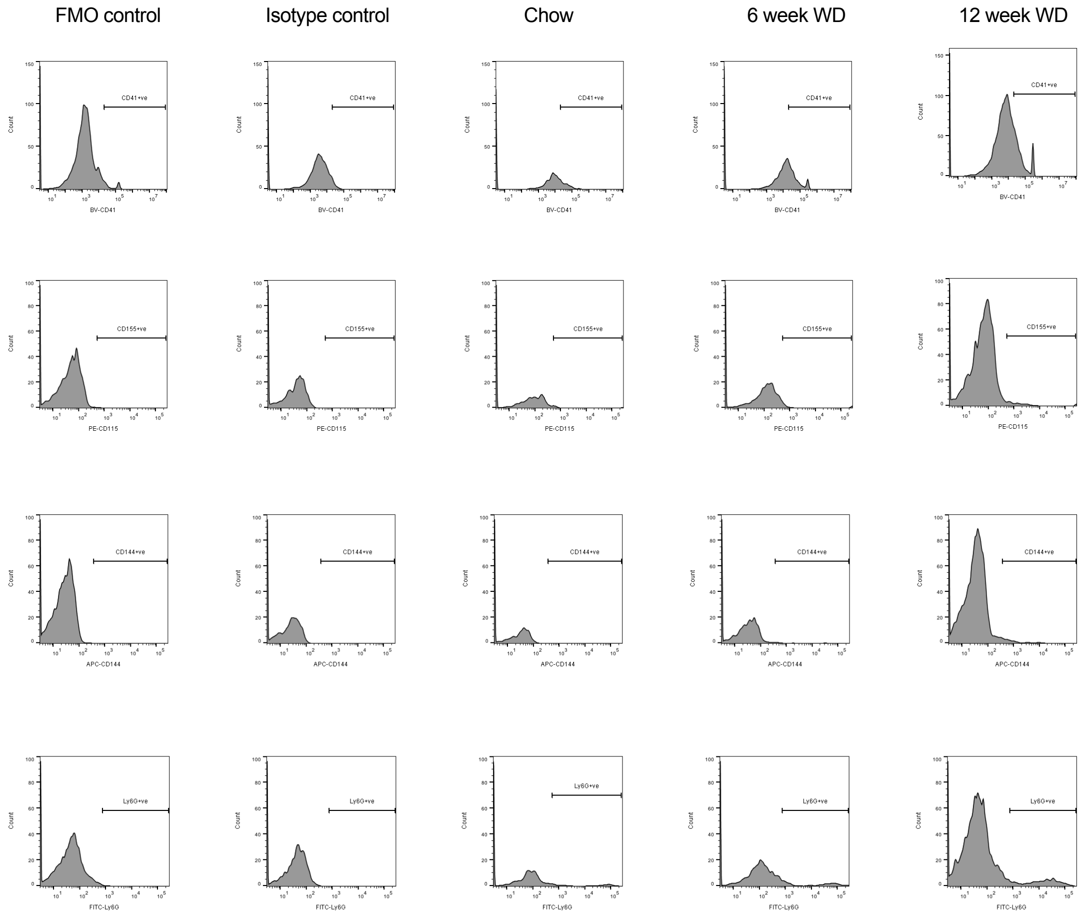

Supplementary Figure 13. Gating strategy for flow cytometry analiysis of MVs present in mouse aortic arch homogentes. Representative flow cytometry histogram plots showing the gating strategy for the identification of $CD41^+$, $CD115^+$, $CD144^+$ and $Ly6G^+$ MVs in mouse samples. Fluorescence minus one samples (containing all fluorochromes except the fluorochome labeling the marker of interest) were used to set the positive gate and isotype control samples were run to ensure the signal was due to specific binding of the antibody to the surface marker. Aortic arch homogenates prepared from *ApoE*$^{-/-}$ mice on chow for 20 weeks or Western diet for 6 or 20 weeks were then incubated with antibodies to all markers. The number of MVs in the positive gate was quantified by running the samples with Sphero™AccuCount counting beads.

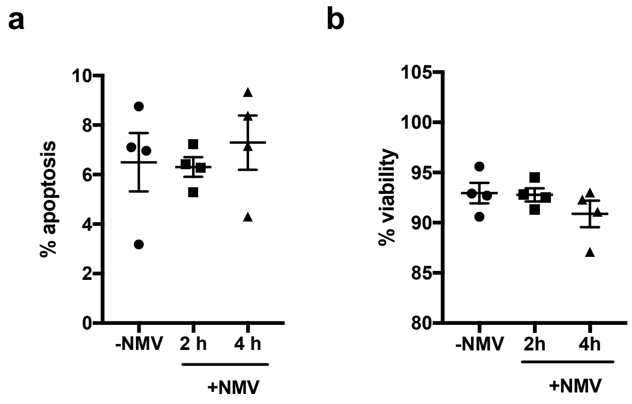

Supplementary Figure 14. Endothelial cell apoptosis and viability. (**a**) Apoptosis and (**b**) necrosis rates in HCAEC were measured with (+NMV) and without (-NMV) incubation with NMVs for 2 and 4 h using a flow cytometry based assay that measured Annexin V binding and propidium iodide uptake. Data are presented as mean ± SEM (n = 4 independent experiments) and statistical significance evaluated using a one-way ANOVA followed by Dunnett's comparing all samples to HCAEC alone (-NMV). Source data are provided as a Source Data file.

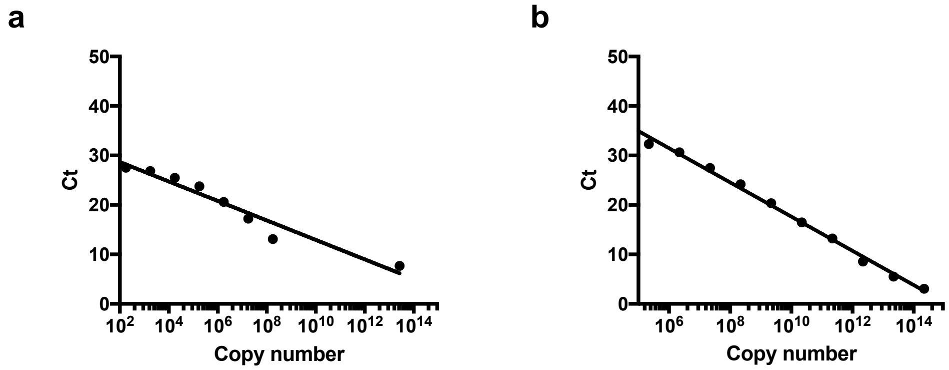

Supplementary Figure 15. Standard curves for miR copy number determination. Standard curves were generated using chemically synthesised RNA oligonucleotides corresponding to the mature miR sequence of (**a**) *miR-155* and (**b**) *miR-223*. The data represents the measured Ct values of each dilution (measured in triplicate). The line indicated is the line of best fit. Source data are provided as a Source Data file.

Supplementary Tables

Meal	Food	Constituents (g)			Energy (kJ)	% of intake
		Protein	Carbohydrate	Fat		
**Breakfast**	4 large pork sausages (230 g)<br>6 rashers of streaky bacon (120 g)<br>2 large fried eggs (120 g)<br>1 slice fried white bread (36 g)<br>whole milk (300 mL)	95	70	105	6715	27
**Lunch**	2 slices of medium white bread (72 g)<br>butter (20 g)<br>mayonnaise (20 g),<br>cheddar cheese (60 g)	22	32	53	2900	12
**Snack**	Potato crisps (50 g)<br>2 medium sausage rolls (120 g)	15	56	37	2582	10
**Dinner**	3 beef burgers (300 g)<br>6 rashers of streaky bacon (120 g)<br>cheddar cheese (90 g)<br>coleslaw (150 g)	135	13	164	8653	35
**Dessert**	Chocolate chip muffin (80 g)<br>double cream (140 mL)	7	41	84	3966	16
**Total**		**274**	**212**	**443**	**24816**	

Supplementary Table 1. Representative example of a day's food intake for human high fat diet studies. In this example diet plan, protein, carbohydrate and fat contributed 18%, 15% and 67% of total energy intake. Saturated fat made up 41% of the ingested fat content.

	Circulating microvesicles μL^{-1} plasma - pre high fat diet			
**Subject**	**CD14 (monocyte)**	**CD41 (platelet)**	**CD66b (neutrophil)**	**CD144 (endothelial cell)**
1	345	3006	3892	1009
2	212	2903	2905	931
3	274	2064	3792	363
4	226	3410	2639	254
5	458	3537	2405	692
6	101	4260	4500	289
7	33	3966	3148	116
8	197	4944	4010	184
9	100	9210	4849	174
10	307	9963	5211	141
11	372	4911	2552	41
12	135	2667	2859	607
13	139	2324	3481	2324
14	119	1998	975	290
15	283	5439	846	1459

Supplementary Table 2. Human plasma microvesicle numbers pre high fat diet. Individual data sets for each subject that took part in the high fat diet study. A week long, high fat diet intervention was carried out in order to increase daily energy intake by approximately 50% (19868 ± 759 kJ). Macronutrient intake was 333 ± 14 g [64%] fat, 188 ± 8 g [16%] protein, and 237 ± 8 g [20%] carbohydrate. Fasting venous blood samples were obtained in the morning before commencing the high fat diet. Blood samples were collected at least 12 hours after consuming the previous evening meal. Platelet poor plasma samples were stained with fluorescent antibodies to surface markers specific to the parent cell and MVs quantified using Sphero™AccuCount beads. The number of events in the positive gate for each marker was analyzed using FlowJo analysis software (Tree star Inc, Ashland, OR) and the total number of MVs in each subpopulation calculated.

	Circulating microvesicles μL^{-1} plasma - post high fat diet			
**Subject**	**CD14 (monocyte)**	**CD41 (platelet)**	**CD66b (neutrophil)**	**CD144 (endothelial cell)**
1	393	3064	4401	2186
2	99	6169	2956	269
3	250	9674	5363	272
4	244	11926	4140	884
5	492	6296	2815	846
6	127	3049	2543	483
7	109	11187	5286	698
8	321	11926	6204	533
9	129	8311	4226	349
10	296	12849	6069	333
11	503	3506	4027	309
12	176	4720	3500	721
13	277	5829	6129	1379
14	341	7364	2331	477
15	245	5928	946	1591

Supplementary Table 3. Human plasma microvesicle numbers post high fat diet. Individual data sets for each subject that took part in the high fat diet study. A week long, high fat diet intervention was carried out in order to increase daily energy intake by approximately 50% (19868 ± 759 kJ). Macronutrient intake was 333 ± 14 g [64%] fat, 188 ± 8 g [16%] protein, and 237 ± 8 g [20%] carbohydrate. Fasting venous blood samples were obtained after 7 days adherence to the diet. Blood samples were collected at least 12 hours after consuming the previous evening meal. Platelet poor plasma samples were stained with fluorescent antibodies to surface markers specific to the parent cell and MVs quantified using Sphero™AccuCount beads. The number of events in the positive gate for each marker was analyzed using FlowJo analysis software (Tree star Inc, Ashland, OR) and the total number of MVs in each subpopulation calculated.

Mouse	Microvesicles μL^{-1} aortic arch homogenate - 20 week chow diet			
	CD41 (platelet)	CD115 (monocyte)	CD144 (endothelial cell)	Ly6G (neutrophil)
1	13	13	5	14
2	18	8	4	17
3	24	11	4	25
4	15	5	3	14
5	19	15	4	30
6	14	7	3	12

Mouse	Microvesicles μL^{-1} aortic arch homogenate - 6 week Western diet			
	CD41 (platelet)	CD115 (monocyte)	CD144 (endothelial cell)	Ly6G (neutrophil)
1	22	14	5	14
2	15	7	3	9
3	19	10	5	29
4	37	23	8	40
5	35	21	7	34
6	28	15	5	17
7	20	15	8	20

Mouse	Microvesicles μL^{-1} aortic arch homogenate - 20 week Western diet			
	CD41 (platelet)	CD115 (monocyte)	CD144 (endothelial cell)	Ly6G (neutrophil)
1	61	24	4	36
2	53	48	4	36
3	57	28	10	71
4	69	26	27	58

Supplementary Table 4. Mouse aortic arch homogenate microvesicle numbers pre and post high fat diet. The aortic arch from mice fed on chow or Western diet (WD) was homogenized. Samples were stained with fluorescent antibodies to surface markers specific to the parent cell and MVs quantified using Sphero™AccuCount beads. The number of events in the positive gate for each marker was analyzed using FlowJo analysis software (Tree star Inc, Ashland, OR) and the total number of MVs in each subpopulation calculated.

	SYBR GREEN	
**gene**	**Forward primer 5′–3′**	**Reverse primer 5′–3′**
β-actin	GCATGGGTCAGAAGGATTCCT	TCGTCCCAGTTGGTGACGAT
BCL6	CGCAACTCTGAAGAGCCACCTG CG	TTTGTGACGGAAATGCAGGTTA
REL-A	TCAAGATCTGCCGAGTGAAC	TGTCCTCTTTCTGCACCTTG
ICAM-1	CACAAGCCACGCCTCCCTGAAC CTA	TGTGGGCCTTTGTGTTTTGATGCT A
VCAM-1	CATTGACTTGCAGCACCACA	AGATGTGGTCCCCTCATTCG
MCP-1	TGGGTTGTGGAGTGAGTGTT	GCAGAAGTGGGTTCAGGATT

	Taqman
Human	
miR-155-5p	UUAAUGCUAAUCGUGAUAGGGGUU
miR-223-3p	UGUCAGUUUGUCAAAUACCCCA
Mouse	
miR-155-5p	UUAAUGCUAAUUGUGAUAGGGGU
miR-223-3p	UGUCAGUUUGUCAAAUACCCCA

Supplementary Table 5. Primers used for RT-qPCR analysis of alterations in gene expression and micro-RNA expression. Details of the primers used as described in the RNA extraction and RT-qPCR section of the Methods.
